# Quantitative ^1^H Nuclear Magnetic Resonance Method for Assessing the Purity of Dipotassium Glycyrrhizinate

**DOI:** 10.3390/molecules26123549

**Published:** 2021-06-10

**Authors:** Yuan-Yuan Zhang, Jie Zhang, Wen-Xuan Zhang, Yue Wang, Ying-Hong Wang, Qing-Yun Yang, Song Wu

**Affiliations:** State Key Laboratory of Bioactive Substance and Function of Natural Medicines, Institute of Materia Medica, Chinese Academy of Medical Sciences & Peking Union Medical College, Beijing 100050, China; zhangyy@imm.ac.cn (Y.-Y.Z.); zhd@imm.ac.cn (J.Z.); wxzhang@imm.ac.cn (W.-X.Z.); wangyue@imm.ac.cn (Y.W.); wyh@imm.ac.cn (Y.-H.W.)

**Keywords:** qNMR, dipotassium glycyrrhizinate, methodology validation, purity

## Abstract

A simple, rapid, accurate, and selective quantitative method based on ^1^H nuclear magnetic resonance (qNMR) was successfully established and developed for assessing the purity of dipotassium glycyrrhizinate (KG). In this study, using potassium hydrogen phthalate and fumaric acid as internal standard (IS), several important experimental parameters, such as relaxation delay and pulse angle, were explored. Reliability, specificity, linearity, limit of quantification, precision, stability, and accuracy were also validated. Calibration results obtained from qNMR were consistent with those obtained from HPLC coupled with ultraviolet detection. The proposed method, independent of the reference standard substance, is a useful, reliable, and practical protocol for the determination of KG and glycyrrhizin analogs.

## 1. Introduction

Quantitative nuclear magnetic resonance (qNMR) is a well-established technique and a powerful tool for the quantitative analysis of organic molecules. In the past few decades, the sensitivity and resolution of NMR technology have been greatly improved; the limits of detection and quantification are less than millimolar concentrations [1]. This method is used widely in pharmaceutics [2], foods [3], proteomics, metabolomics [4], and natural products [5]. The qNMR method has special advantages compared with classical chromatography because the signal area in the NMR spectrum is directly proportional to the number of nuclei giving rise to specific resonance [6]. This method not only reveals structural information, but is also independent of varying physical properties and has relatively easy sample preparation, a non-destructive test for samples, and so on [7,8]. The qNMR technique is superior for the calibration of the reference standard based on quantitative estimation without relying on targeted authentic references [9].

**Figure 1:**
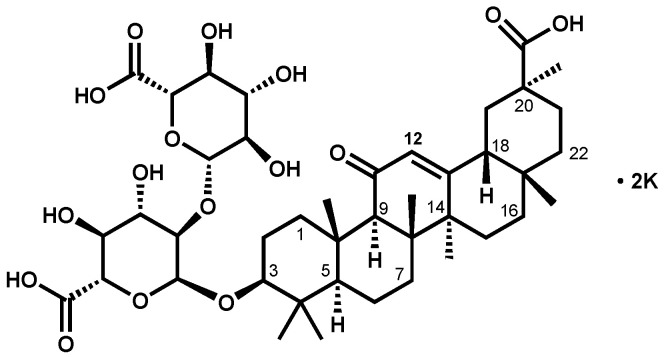
Structure of dipotassium glycyrrhizinate (KG).

Glycyrrhizin, a compound obtained from *Licorice* root [10], is widely used to treat liver diseases, such as hepatitis B, hepatitis C, liver fibrosis, and liver cirrhosis [11,12,13,14]. Glycyrrhizin and its derivatives, such as diammonium glycyrrhizinate, sodium glycyrrhizate, and dipotassium glycyrrhizinate, are made into injections, capsules, granules, and oral solutions for clinical applications [15]. Dipotassium glycyrrhizinate (KG), [(3*β*,20*β*)-20-carboxy-11-oxo-30-norolean-12-en-3-yl-2-*O*-*β*-d-glucopyranuronosyl-*α*-d-glucopyranosiduronic acid, potassium salt (1:2)] is a dipotassium salt of glycyrrhizin (Figure 1). This compound is mainly applied as an anti-inflammatory [16], antitumor [17], antivirus [18], and skin-conditioning agent for cosmetics [19] because of its chemical stability and good solubility. Analytical methods for glycyrrhizic acids mainly include chromatography methods, such as thin-layer chromatography (TLC) [20], high-performance liquid chromatography (HPLC) [21], and polargraph [22]. However, a common characteristic of glycyrrhizic acids is that most molecular ultraviolet absorption occurs toward the end of the ultraviolet region; the UV absorption of this type of compound generally weak. Thus, calibration of the reference standard of glycyrrhizic acids is difficult using chromatographic analysis.

This study proposes an accurate, specific, structure-reflecting, and reproducible method for the calibration of reference standards of KG by using qNMR spectroscopy as a substitute to traditional methods. Although the qNMR method has a certain advantage in determining the absolute content of a sample, relevant reports on determining glycyrrhizic acids by qNMR are still scarce. The proposed method used potassium hydrogen phthalate (KHP) and fumaric acid (FA) as the internal standard (IS), 2,2,3,3-d-(4)-3-trimethylsilyl propionic acid sodium salt (TSP) as internal standard of chemical shift (at *δ* 0 ppm), and deuterium oxide (D_2_O) for subsequent dilutions. Three batches of KG reference standards were chosen to verify the accuracy of the proposed qNMR method. The calibration results were further compared with those obtained by the HPLC-UV method. Results indicated that qNMR is a reliable method for the purity analysis of KG and glycyrrhizin analogs.

## 2. Results and Discussion

### 2.1. Selection of Quantification Signal from Analytes

**Figure 2:**
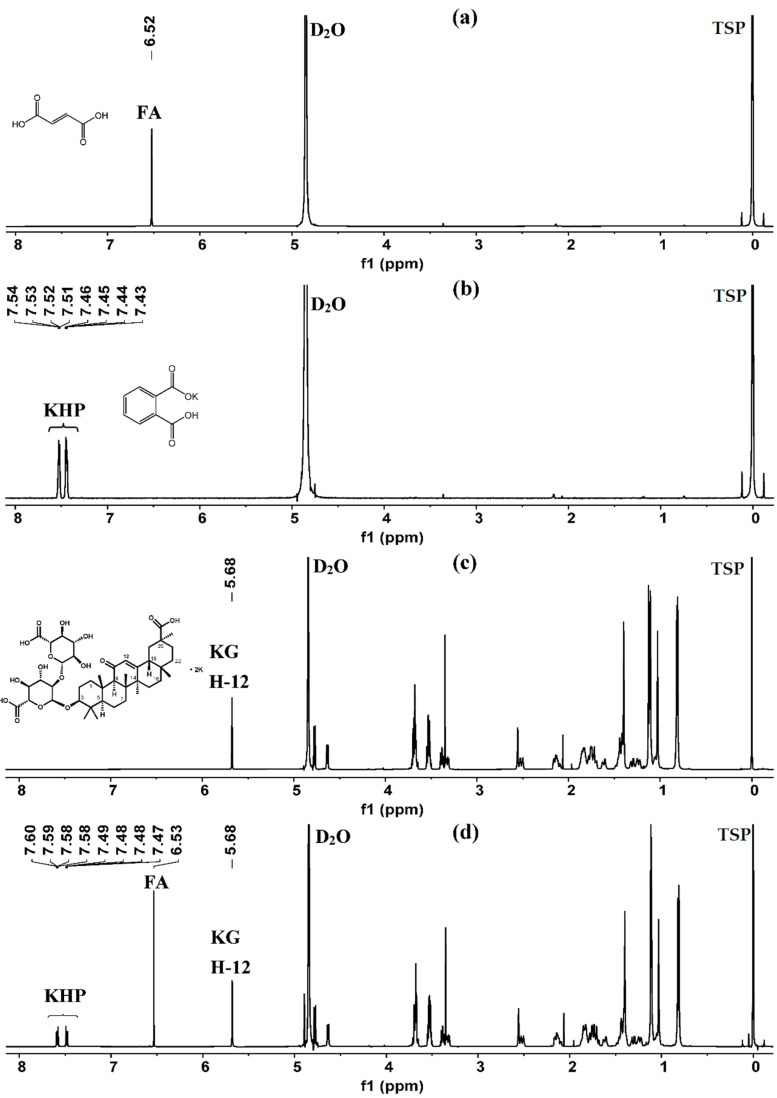
(**a**) ^1^H-NMR spectrum of fumaric acid (FA) in D_2_O; (**b**) ^1^H-NMR spectrum of potassium hydrogen phthalate (KHP) in D_2_O; (**c**) ^1^H-NMR spectrum of dipotassium glycyrrhizinate (KG) in D_2_O; (**d**) ^1^H-NMR spectrum of FA, KHP, and KG in D_2_O (500 MHz).

Protons on skeletons, rather than sugar moiety or substituent groups, are preferred as quantitative protons because signals arising from the skeleton are constant, less easily interfered, and indicate better commonality [23]. Furthermore, appropriate quantification signals need to meet several criteria, including good separation with other signals and good line shape. Figure 2 shows that most proton signals from the skeleton are present upfield as multiplets. These signals cannot be chosen as quantification signals because they are complicated and overlapping. The ^1^H-NMR spectrum of KG shows a characteristic signal at *δ* 5.68 ppm (1H, s, 12-H), belonging to the hydrogen proton on the olefinic carbon atom (C-12). The baseline is flat and straight within the range of 5.0–8.0 ppm. The H-12 signal occurs downfield with good separation and line shape. In addition, the signal forms a single peak because of a lack of protons coupling with it. As a consequence, this signal was selected as the quantification signal.

### 2.2. Selection of Solvent and Internal Standard

Several deuterated solvents were screened for the experiment. Deuterium oxide (D_2_O) was an excellent solvent because it ensured good solubility of KG and KHP. FA can be dissolved very well in D_2_O at 50 °C. The quantitative signals of KG and IS in D_2_O did not overlap with other signals. Moreover, D_2_O is an economical and environmentally-friendly solvent.

In this study, KHP and FA were investigated as IS; both of them were soluble in D_2_O. FA had a good quantification signal at *δ* 6.52 ppm (2H, s). Nevertheless, the relaxation time of protons on the ethylenic group is longer, and the D_1_ value needs to be set at least more than 30 s to ensure that protons are fully relaxed to obtain accurate results. The signals at *δ* 7.47–7.49 ppm (2H, m) and *δ* 7.58–7.60 ppm (2H, m) from aromatic protons of KHP were used as the quantification signal, which were both convenient and accurate for quantification, although the peak shape is not good and the coupling is complex. None of the above quantitative signals from different IS overlapped. In comparison, the single peak at *δ* 6.52 ppm was close to the quantification signal at *δ* 5.68 ppm, resulting in a good quantification result. Therefore, KHP and FA were used as the IS in all the experiments of method verification and sample analysis.

### 2.3. Optimization of Experiment Parameters

Relaxation delay (D1) and pulse angle are important parameters for accurate quantification, and are correlated with each other. In general, maximum sensitivity will be obtained at a 90° pulse angle and D1 was required to be five times greater than the longest longitudinal relaxation time (T1) of the quantification protons; at a 30° pulse angle, D1 should be more than 7/3 times of T1 [7, 24]. A 30° pulse angle was adopted in this study to avoid time consumption and inefficiency. According to Bruker’s T1ir pulse program, the T1s of FA and KHP protons were 10.390 s and 3.713 s, respectively. The T1 of H-12 from KG was 1.129 s (Supplemental Materials Figure S1). Therefore, D1 should be set to more than 25 s, which was further proved in subsequent experiment. The area ratio of KG versus FA and KHP protons was determined from their ^1^H NMR spectra by using different D1 values. For D1 of 1, 2, 4, 8, 16, 32, 48, 64, and 70 s, the integral areas for FA were 1.69, 1.68, 1.68, 1.75, 1.77, 1.79, 1.79, 1.79, and 1.79, respectively. As shown in Table S1, the peak areas of FA increased with increasing D1, and they became invariant as the D1 was increased from 1 s to 32 s. Therefore, D1 was set as 30 s in subsequent experiments. The number of scans is also an important parameter, which is closely related to *S/N* values. In this experiment, the number of scans was set to 8, 16, and 32. The results show that 16 scans could ensure *S/N* values above 150 [23].

### 2.4. Method Validation

#### 2.4.1. Specificity

The ^1^H-NMR spectra of KG, FA, KHP, and their mixture are shown in Figure 2. The baseline was flat and straight in the range above 5.5 ppm, where no other signals are present except for the quantification signals. The signals at *δ* 5.68 ppm (H-12, KG), *δ* 6.52 ppm (FA), *δ* 7.43 ppm, and 7.50 ppm (KHP) were well separated and did not overlap with one another. The *S*/*N* values of these signals were above 800.

#### 2.4.2. Linearity and Range

Linearity was checked by preparing standard solutions at six levels using FA, KHP, and KG in different molar ratios (Table 1). A linearity curve was plotted and the correlation coefficient obtained from the linear regression curves were 0.9996 and 0.9996 when FA and KHP were used as IS, respectively. The linear regression yielded a regression line of y = 0.0571x + 0.0237 and y = 0.0503x + 0.0207. The results indicated that the established method had good linearity over the concentration ranges (*w*/*w*) from 4.02 to 30.37 and 3.96 to 29.93.

#### 2.4.3. Limit of Quantification (LOQ)

Malz and Jancke [24] reported that the *S*/*N* ratio should be greater than 150:1 for ^1^H-NMR to achieve accurate quantification with uncertainly of 1%. LOQ was assessed by determining the *S*/*N* ratio and was investigated using the analyte with the minimum concentration (1.02 mg·mL^−1^) of KG. In addition, the lowest *S*/*N* ratio was 249.06.

#### 2.4.4. Precision and Stability

The precision of the method was estimated by performing six replicate measurements of the mixture containing KG and IS (FA and KHP). As shown in Table 2, the relative standard deviation (RSD) values of precision were 0.31% and 0.24%, when FA and KHP were used as IS, respectively. Reproducibility was evaluated by analysis of six different solutions with the same concentration independently prepared with KG and IS (FA and KHP). The RSD values were 0.67% and 0.57%, indicating good precision and high reproducibility.

The stability of the same sample solution was observed at different time points within 24 h. The RSD values were within 0.5%, confirming that the sample solution had good stability in the NMR sample tube within 24 h at ambient temperature.

#### 2.4.5. Accuracy 

Recovery test was used to evaluate the accuracy of the proposed qNMR method. The average recovery rates were 99.29% and 99.90% when FA and KHP were used as IS, and the RSD values were within 2.0%. Detailed data are shown in Table 3. The results suggest that the proposed method has good accuracy.

#### 2.4.6. Robustness

Several important acquisition parameters were investigated stepwise in wide ranges to evaluate the robustness of the proposed method (Table 4). The purity levels of KG measured with the optimal parameters were 97.02% and 97.94% when using FA and KHP as IS, respectively. The results indicated that these parameters did not have significant influences on the quantification results, including in relaxation delay (D1), time domain (TD), scan number (NS), and pulse length (P1). For instance, variation within 50% of NS and TD did not significantly alter the results, with the maximum difference values below 1%. D1 has obvious effects on the quantification results, and an improperly set value of D1 would introduce an error in quantification due to incomplete relaxation of longitudinal magnetization [24]. In this study, D1 was required to be at least 30 s because the T1s of FA protons was 10.39 s.

### 2.5. Analysis Results by qNMR and HPLC

The established analytical method was utilized for the calibration of three batches of KG samples and compared with conventional HPLC-UV analysis. As previously mentioned, KHP and FA were used as the IS in the proposed qNMR method. As shown in Table 5, no significant differences were found between the quantitative results from the HPLC-UV detection system and those obtained by qNMR method. Multiple analytes contain nondetectable residual water and the inorganic element potassium, which did not have any response on the UV detector. As such, determination of the mass percentage of water and potassium element were conducted using Karl Fisher titrimetry and inductively coupled plasma-optical emission spectroscopy (ICP-OES). Absolute content was determined directly by the qNMR method. The results showed that the qNMR technique could be a practical, reliable, and objective method for testing the purity of glycyrrhizin and its derivatives.

## 3. Experimental Section

### 3.1. Materials

Dipotassium glycyrrhizinate (KG, batch No. 8060633, 8070201, 9010291) was synthesized in our laboratory. Fumaric acid (FA) reference standard (purity 99.9%, standard for qNMR) was purchased from the Chinese National Institutes for Food and Drug Control (NIFDC, China, cat. No. 111541-201803). Potassium hydrogen phthalate (KHP, purity 99.95%, standard for qNMR) was obtained from the National Institute of Metrology, China (Beijing, China, code GBW(E)060019). Deuterated solvent (D_2_O, 99.9%) was acquired from 3A Chemicals (Shanghai, China). 2,2,3,3-d-(4)-3-Trimethylsilyl propionic acid sodium salt (TSP) was supplied by Cambridge Isotope Laboratories, Inc. (Andover, MA, USA). Ammonium glycyrrhizinate (AG) reference standard (purity 96.2%, standard for HPLC) was provided by the Chinese National Institutes for Food and Drug Control (NIFDC, Beijing, China, cat. No.110731-202021). HPLC-grade acetonitrile and methanol were purchased from Mallinckrodt Baker Inc. (Phillipsburg, NJ, USA) and used for preparation of mobile phases. Wahaha purified water was used. The standard stock solution of K (200 mg·L^−1^) was obtained from National Center of Analysis and Testing for Nonferrous Metals and Electronic Materials (Beijing, China). Deionized water was prepared from a Milli-Q water purification system (Millipore Corporation, Bedford, MA, USA). Hydranal^®^-Composite 5 (Honeywell, Fluka, Germany) was used as the titrant for Karl-Fischer titration.

### 3.2. Instrument

All ^1^H-NMR spectra were obtained at 298.0 K by using a Bruker Avance Spectrometer at 500.06 MHz proton frequency (AV-III-500, Burlingame, CA, USA). qNMR experiments were performed with the following optimized parameters: pulse angle, 30; pulse width, 2.4 μs; data points, 64 K; number of scans, 16; acquisition time (AQ), 7.27 s; spectral width (SW), 0.230 Hz. All spectra were processed using Bruker’s Topspin software (version 3.0, Bruker Biospin, Spring, TX, USA). A line-broadening factor of 0.3 Hz was applied to FIDs before Fourier transformation. The repetition delay was 30 s, which was calculated using the inversion recovery pulse program. All chemical shifts were reported in parts per million (ppm) relative to TSP at 0.00 ppm. Each measurement was repeated three times for statistical analysis. The values of T1 relaxation time for targeted protons were listed as follows: for KG, *δ* 5.68 ppm (H-12), T1 = 1.129 s; for FA, *δ* 6.52 ppm, T1 = 10.39 s; for KHP, *δ* 7.47–7.60 ppm, T1 = 3.713 s.

HPLC analysis was performed using a Shimadzu liquid chromatography UFLC-20ADXR equipped with an SPD-M20A spectrophotometric detector (Shimadzu Co., Kyoto, Japan). LC separation was achieved using an YMC C18 column (250 mm × 4.6 mm I.D.; 5 μm particle size) maintained at 30 °C. The mobile phase was a mixture of 0.05% phosphoric acid (mobile phase A) and acetonitrile (mobile phase B) in a constant proportion of 55:45 (*v*/*v*) at a flow rate of 0.7 mL/min. The injection volume was set at 10 μL, and UV detection was conducted at 250 nm.

The mass percentage of the concentration of potassium was determined by Agilent 5800 ICP-OES instrument (Agilent Technologies, Santa Clara, CA, USA). The operating conditions for the elemental analysis are presented in Supplemental Materials Table S2. All analytes were measured in the radial observation mode and the signals were integrated for 10 s.

Moisture measurements were performed with Karl Fischer Moisture Titrator model V20 (Mettler Toledo Instruments Inc., Greifensee, Switzerland). The measurements were corrected for the deviation of the standard from the theoretical water. All the samples were weighed on a Mettler Toledo AB 135-S balance (0.01 mg, Greifensee, Switzerland).

### 3.3. Sample Preparations and Calculations

#### 3.3.1. qNMR Analysis of Analytes

About 10 mg of FA was accurately weighed, dissolved in 5.0 mL of D_2_O at 50 °C, and statically cooled to room temperature. About 10 mg of KHP was accurately weighed and dissolved into the FA solution. The mixed IS solution of FA and KHP was produced at a concentration of 1 mg·mL^−1^ by using D_2_O. Approximately 10 mg of KG was accurately weighed, transferred into a 2.0 mL stoppered tube, and then added with 2 mg of TSP. The mixture was dissolved in 1 mL of D_2_O and added with 500 μL of the IS solution and appropriate amounts of D_2_O. The solution was vortexed for 30 s until the sample was completely dissolved. The solution was transferred into 5-mm NMR tubes, and the spectrum was obtained (Supplemental Materials Figures S2–S5). All the samples were prepared in triplicate, and the average value was used for calculation.

The purity of analyte *P_x_* was calculated using Formula (1) [23]. The results are shown in Table 5.

(1) $$P_{x}=\frac{A_{x}}{A_{std}}\frac{N_{std}}{N_{x}}\frac{M_{x}}{M_{std}}\frac{m_{std}}{m_{x}}\times P_{std}\times 100\%$$

where *A_x_* is the integral value of the signal that belongs to KG, *A_std_* is the integral value of the signal that belongs to FA or KHP, *N_std_* and *N_x_* correspond to the number of spins of IS (FA, *N* = 2; KHP, *N* = 4) and KG (*N* = 1), respectively, *M_x_* and *M_std_* are the molecular weight of KG (899.1) and IS (FA, *M* = 116.1; KHP, *M* = 204.2), respectively, *m_std_* and *P_std_* are the weighted mass and the purity of IS, respectively, and *m_x_* is the weighted mass of KG (mg).

#### 3.3.2. qNMR Method Validation

The qNMR method was validated in terms of linearity, precision, repeatability, stability, accuracy, and robustness. For method validation, all ^1^H-NMR experimental samples were dissolved in D_2_O. FA and KHP were dissolved in D_2_O (2 mg·mL^−1^) as the mixed IS solution. To establish linearity, we prepared a calibration plot by analyzing six solutions in the concentration ranges (*w*/*w*) of 4.02–30.37 (*m*_KG_/*m*_FA_) and 3.96–29.93 (*m*_KG_/*m*_KPH_), as shown in Table 1. The intercept, slope, and correlation coefficient were determined by linear regression. The precision of the proposed qNMR method was assessed by six replicated measurements of the mixture containing KG and IS at the concentrations (*w*/*w*) of 20.51 (*m*_KG_/*m*_FA_) and 20.33 (*m*_KG_/*m*_KPH_). Solution stability was evaluated by analyzing the mixture at different time intervals (0, 1, 2, 4, 8, 12, and 24 h) at room temperature. Reproducibility was evaluated by analysis of six different solutions with the same concentrations of KG and IS. Each sample was measured three times, and the results were estimated by calculating RSD%. Detailed data are shown in Table 2. The accuracy of the qNMR method was determined by the recovery test. In this study, known quantities of KG (at 80%, 100%, and 120%) were added into the same quantity of the analyte. The assay at each concentration level was performed in triplicate. Recovery was calculated by comparison of the experimental and theoretical values (Table 3).

#### 3.3.3. HPLC Analysis of Analytes

A total of 10 mg of KG was dissolved in 5 mL of 70% ethanol/water solution and diluted to 10 mL. For 1.0 mL of the solution, 70% ethanol/water solution was added and diluted to 10 mL. The solution was used as the sample solution. Standard solution was prepared using the same method with AG as the reference standard. The sample and standard solutions were filtered through a 0.45 μm filter. The chromatographic peak area was used to calculate the purity of each sample (Supplemental Materials Figures S6 and S7). The amount of glycyrrhizic acid *P_x_* was calculated using Formula (2):

(2) $$P_{x}=\frac{A_{x}}{A_{s}}\frac{m_{s}}{m_{x}}\frac{M_{KG}}{M_{AG}}\times P_{s}\times 100\%$$

where *A_x_* and *A*_s_ are the peak response from sample and standard solutions, *m*_s_ and *m_x_* are the weighted masses of AG reference standard and KG, respectively, *M_KG_* and *M_AG_* are the molecular weights of the KG (899.11) and AG reference standards (839.96), respectively, and *P_s_* is the purity of the AG reference standard.

#### 3.3.4. Potassium Analysis by ICP-OES

For the preparation of calibration curves, the serial dilution method was used to prepare calibration solutions at 10, 50, 100, 150, and 200 mg·L^−1^ from the potassium standard solution (200 mg·L^−1^). The linear correlation coefficient was 0.99997. The linear regression analysis yielded the regression line of y =790.2346x + 356.7248. The calibration curve of K is shown in Supplementary Material Figure S8.

About 100 mg of KG was accurately weighed and transferred into a 50 mL volumetric flask. The flask was filled with 20 mL of deionized water until the sample was completely dissolved. The solution was diluted with deionized water for volume, and the diluted solution was used as the sample solution. All the sample solutions were prepared in triplicate. The external standard curve method was used to calculate the mass percentage of potassium in the KG samples.

## 4. Conclusions

In this study, a simple, rapid, and selective qNMR method was successfully developed and validated for assessment of the purity of KG. Using KHP and FA as IS, key experimental parameters, such as D1, pulse angle, NS, and TD, were explored and optimized. The reliability and accuracy of the qNMR method as well as the feasibility of the two ISs were confirmed through comprehensive method verification. Furthermore, the verification of specificity, linearity, limit of quantification (LOQ), precision, and stability indicated that the qNMR method was simple, rapid, and selective. A comparison of the qNMR method with the HPLC approach revealed that the two methods were almost identical. The calibration results of the three batches of KG samples were consistent with those obtained from high-performance liquid chromatography coupled with ultraviolet detection. Based on the key characteristics of the NMR spectra, the signal intensity is directly proportional to the number of protons responsible for the peak. Hence, the proposed qNMR method is an absolute quantification method that does not require self-reference standard substance. This method could be used as an effective and practical tool for the purity assessment of KG and glycyrrhizin analogs.

## Figures and Tables

**Table 1:** Linearity and range of dipotassium glycyrrhizinate by qNMR.

No.	*m_FA_* (mg)	*m_KHP_* (mg)	*m_KG_* (mg)	*m_KG_*/*m_FA_*	*m_KG_*/*m_KHP_*	*A_KG_*/*A_FA_*	*A_KG_*/*A_KHP_*
1	0.51	0.52	2.04	4.02	3.96	0.24	0.21
2	0.51	0.52	3.06	6.02	5.94	0.36	0.32
3	0.51	0.52	5.22	10.27	10.12	0.62	0.54
4	0.51	0.52	10.55	20.77	20.47	1.22	1.06
5	0.51	0.52	12.56	24.72	24.36	1.44	1.25
6	0.51	0.52	15.43	30.37	29.93	1.74	1.52
Linear equation						y = 0.0571x + 0.0237	y = 0.0503x + 0.0207
*R* ^2^						0.9996	0.9996

**Table 2:** Precision, reproducibility, and stability of dipotassium glycyrrhizinate by qNMR.

	No.	*m_FA_* (mg)	*m_KHP_* (mg)	*m_KG_* (mg)	*P_x_* (%) *	
					FA as IS	KHP as IS
Precision (n = 6)	1	0.51	0.51	10.44	96.67	98.09
	2	0.51	0.51	10.44	97.20	97.63
	3	0.51	0.51	10.44	97.20	98.09
	4	0.51	0.51	10.44	97.63	98.06
	5	0.51	0.51	10.44	97.20	98.09
	6	0.51	0.51	10.44	97.20	97.63
	Average value	/	/	/	97.18	97.93
	RSD%	/	/	/	0.31	0.24
Reproducibility	1	0.52	0.51	10.47	95.88	96.45
	2	0.52	0.51	10.13	96.14	95.81
	3	0.52	0.51	10.40	96.65	96.49
	4	0.52	0.51	10.54	96.62	96.84
	5	0.52	0.51	10.12	95.48	96.04
	6	0.52	0.51	10.14	97.30	97.35
	Average value	/	/	/	96.34	96.50
	RSD%	/	/	/	0.67	0.57
Stability	0 ^#^	0.51	0.51	10.44	97.30	97.66
	1	0.51	0.51	10.44	97.30	97.66
	2	0.51	0.51	10.44	96.86	97.23
	4	0.51	0.51	10.44	98.16	98.06
	8	0.51	0.51	10.44	97.73	97.63
	12	0.51	0.51	10.44	97.73	97.63
	24	0.51	0.51	10.44	98.16	98.06
	Average value	/	/	/	97.60	97.70
	RSD%	/	/	/	0.49	0.29

* *P_x_* (%) is the purity of analyte; ^#^ time in h; /: blank cell.

**Table 3:** Recovery of dipotassium glycyrrhizinate by qNMR.

No.	Amount Added (mg)	FA as IS		KHP as IS	
		Amount Found (mg)	Recovery (%)	Amount Found (mg)	Recovery (%)
1	7.93	7.89	99.49	8.00	100.83
2	7.93	7.88	99.31	8.00	100.83
3	7.93	7.92	99.80	8.05	101.51
4	10.34	10.21	98.79	10.39	100.75
5	10.34	10.22	98.80	10.37	100.49
6	10.34	10.18	98.46	10.32	99.96
7	12.02	12.06	100.33	11.80	98.23
8	12.02	11.89	98.94	11.74	98.23
9	12.02	11.98	99.71	11.83	98.23
Average value (%)	/	/	99.29	/	99.90
RSD (%)	/	/	0.60	/	1.31

**Table 4:** Results of the robustness for the qNMR analysis.

Parameters	Variation	FA as IS		KHP as IS	
		P_x_ (%)	*Diff* (%)	P_x_ (%)	*Diff* (%)
Relaxation delay (D1)	1 s	106.06	9.04	100.30	2.36
	**30 s** ^#^	97.02	/	97.94	/
	70 s	97.67	0.65	98.05	0.11
Time domain (TD)	32 k	97.58	0.56	97.64	0.30
	**64 k**	97.02	/	97.94	/
Number of scans (NS)	8	97.30	0.28	97.66	0.28
	**16**	97.02	/	97.94	/
	32	97.69	0.67	98.08	0.14
Pulse length (Pl)	2.2 μs	97.62	0.60	97.80	0.14
	**2.4 μs**	97.02	/	97.94	/
	2.6 μs	97.47	0.45	97.50	0.44

^#^ Bold values represent standard parameter sets.

**Table 5:** Determination results of the purity test.

Batch No.	qNMR Method (n = 6)			HPLC Method		K% *	Water% ^#^
	IS	%	RSD%	%	RSD%		
8060633	FA	99.27	0.30	99.61	0.23	9.48	2.25
	KHP	99.42	0.27				
8070201	FA	99.59	0.29	99.38	0.50	8.19	2.38
	KHP	99.20	0.27				
9010291	FA	97.18	0.31	97.22	0.34	9.47	3.59
	KHP	97.93	0.24				

* Mass percentage of K; ^#^ mass percentage of water.

## Data Availability

The data presented in this study are available on request from the corresponding author.

## Supplementary Materials

The following are available online at Figure S1: T1 relaxation times of KG, FA, and KHP; Figure S2: ^1^H-NMR spectrum (500 MHz, D_2_O) of KG; Figure S3: ^1^H-NMR spectrum (500 MHz, D_2_O) of FA; Figure S4: ^1^H-NMR spectrum (500 MHz, D_2_O) of KHP; Figure S5: ^1^H-NMR spectrum (500 MHz, D_2_O) of the mixture of FA, KHP and KG; Figure S6: HPLC chromatogram of ammonium glycyrrhizinate reference substance; Figure S7: HPLC chromatogram of KG sample (batch no. 8060633; Figure S8: Calibration curve of K by ICP-OES; Table S1: Integral areas of the internal standards (KHP and FA) and KG at different D1; Table S2: Information on ICP-OES operating conditions.
