# Supplementary material for: Quantitative 1H Nuclear Magnetic Resonance Method for Assessing the Purity of Dipotassium Glycyrrhizinate
Source: Molecules. 2021 Jun 10;26(12):3549. doi: 10.3390/molecules26123549 (PMC8230393; doi:10.3390/molecules26123549)
Supplement: Supplementary file 1 [file molecules-26-03549-s001.zip › molecules-1245045-supplementary.pdf]

Supporting information

Quantitative ^{1}H Nuclear Magnetic Resonance Method for Assessing the Purity of Dipotassium Glycyrrhizinate

Yuanyuan Zhang[#], Jie Zhang[#], Wenxuan Zhang, Yue Wang, Yinghong Wang, Qingyun Yang[*], Song Wu[*]

State Key Laboratory of Bioactive Substance and Function of Natural Medicines, Institute of Materia Medica, Chinese Academy of Medical Sciences & Peking Union Medical College, Beijing 100050, China

Table of Contents

1. Figure S1. T_1 relaxation times of KG, FA and KHP..……,……………………….……....…S1
2. Figure S2. ^{1}H-NMR spectrum (500 MHz, D_2O) of KG ……………….…,….…….……….....…S1
3. Figure S3. ^{1}H-NMR spectrum (500 MHz, D_2O) of FA…………………,………….……......…S2
4. Figure S4. ^{1}H-NMR spectrum (500 MHz, D_2O) of KHP………………,…….……….……....…S2
5. Figure S5. ^{1}H-NMR spectrum (500 MHz, D_2O) of mixture of FA, KHP and KG………........…S3
6. Figure S6. HPLC chromatogram of ammonium glycyrrhizinate reference substance………... S3
7. Figure S7. HPLC chromatogram of KG sample (batch no. 8060633) …….……………..…... S4
8. Figure S8. Calibration curve of K by ICP-OES………………………,…….………….....…S4
9. Table S1. The integral areas of the internal standards (KHP and FA) and KG at different D1..S5
10. Table S2. Information on ICP-OES operating conditions……...………,…….………….....…S5

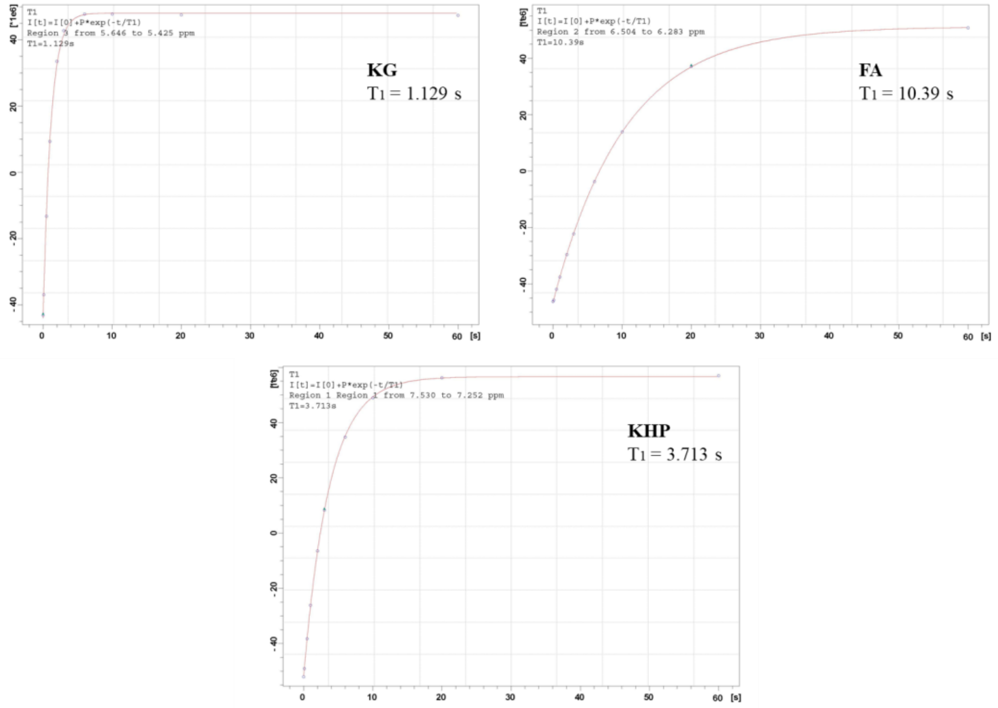

Figure S1. T_1 relaxation times of KG, FA and KHP.

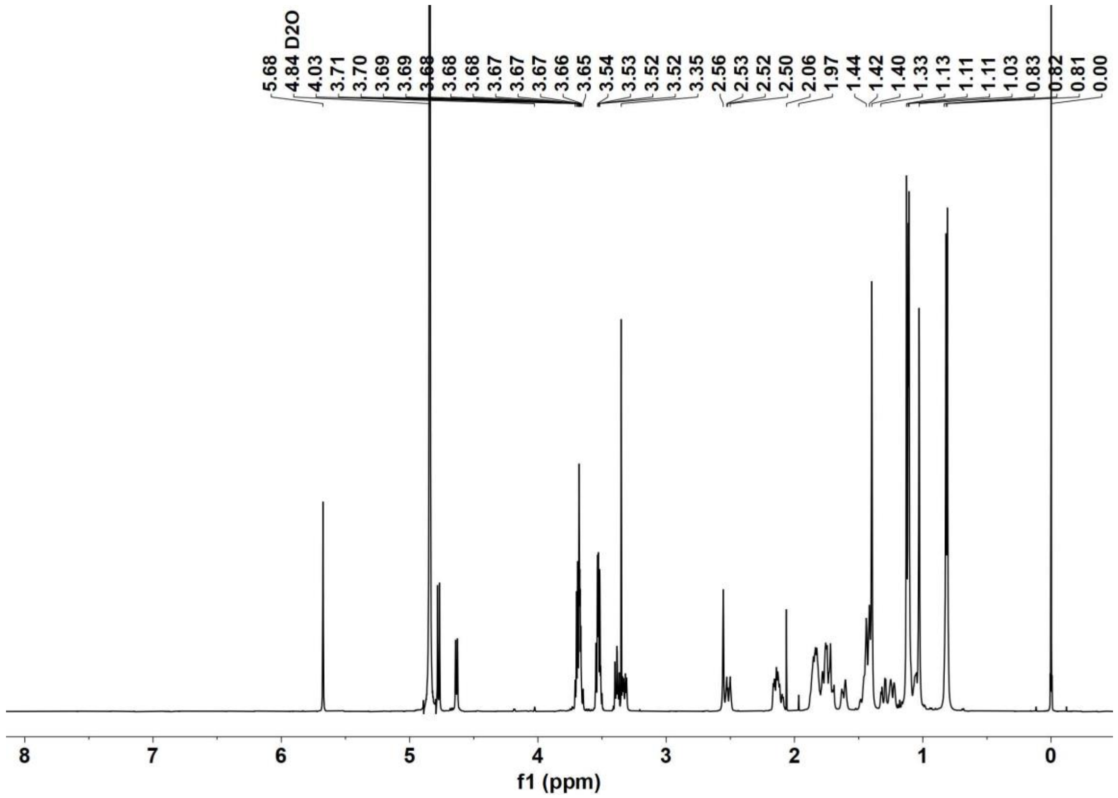

Figure S2. ^{1}H-NMR spectrum (500 MHz, D_2O) of KG

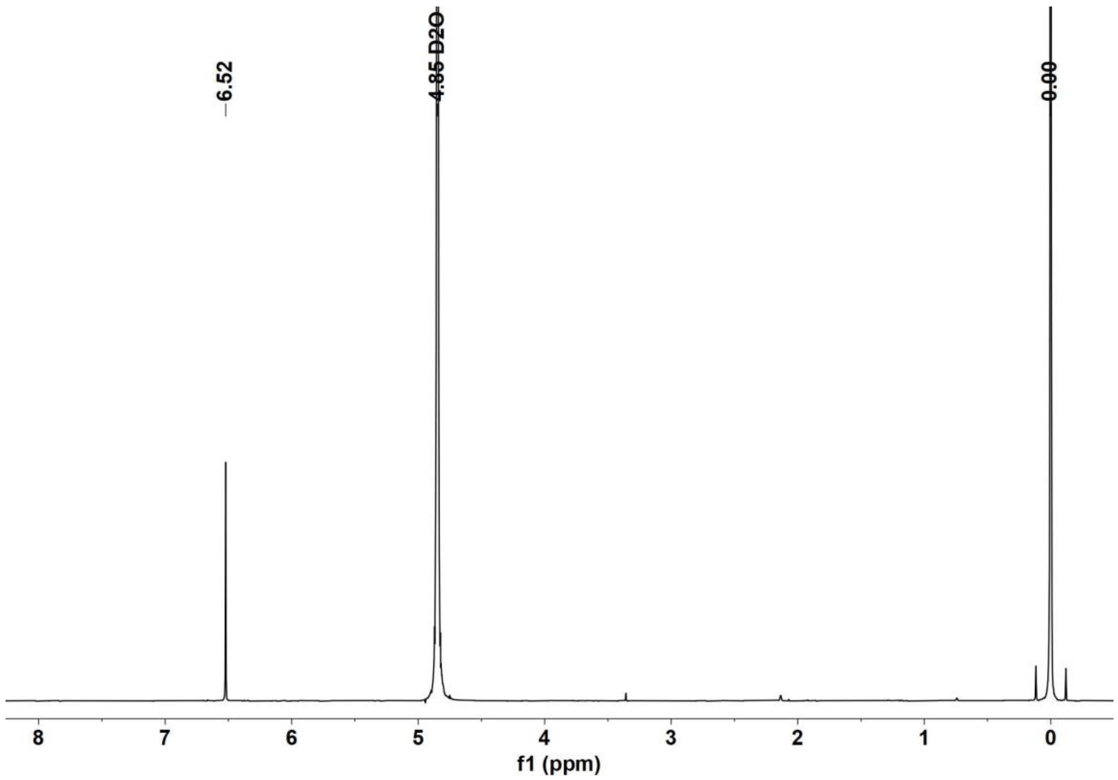

Figure S3. ^{1}H-NMR spectrum (500 MHz, D_2O) of FA

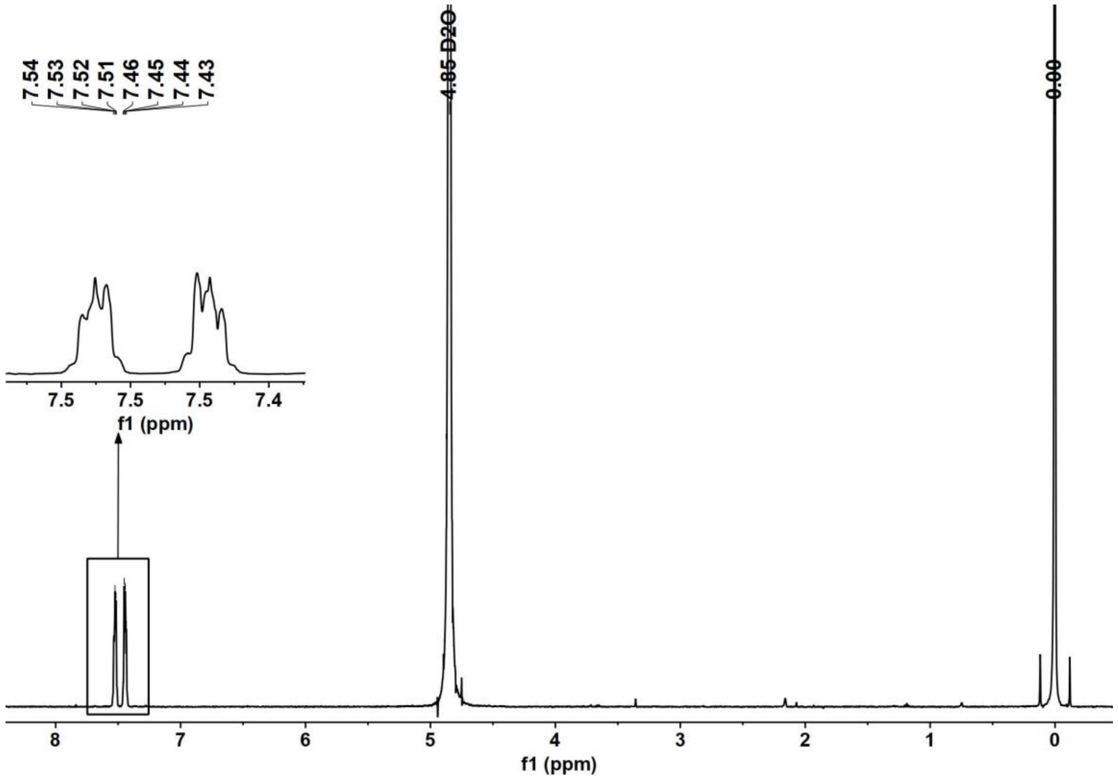

Figure S4. ^{1}H-NMR spectrum (500 MHz, D_2O) of KHP

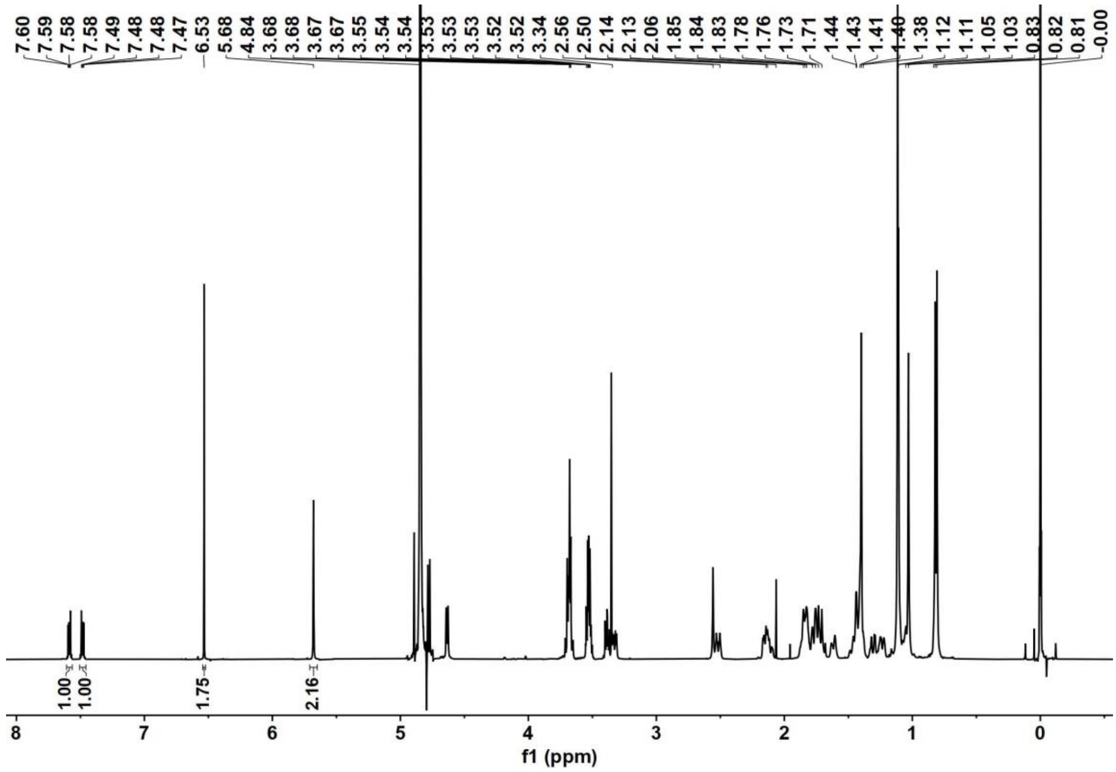

Figure S5. ^{1}H-NMR spectrum of the mixture of FA, KHP and KG in D_2O (500 MHz).

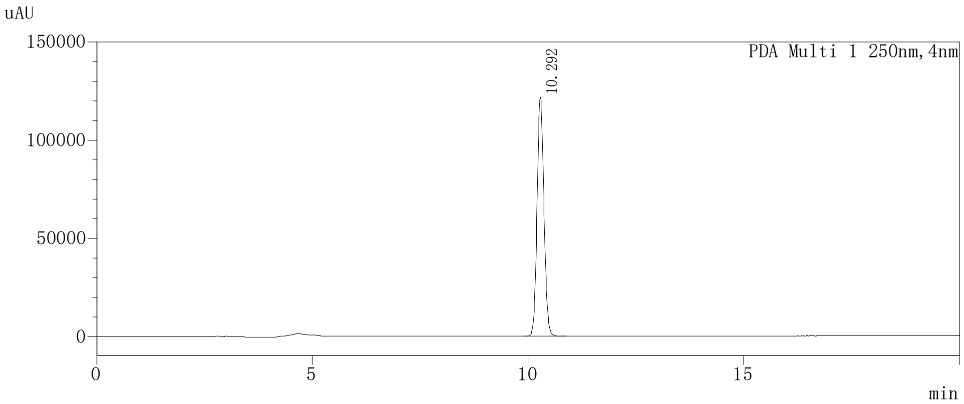

Figure S6. HPLC chromatogram of ammonium glycyrrhizinate reference substance

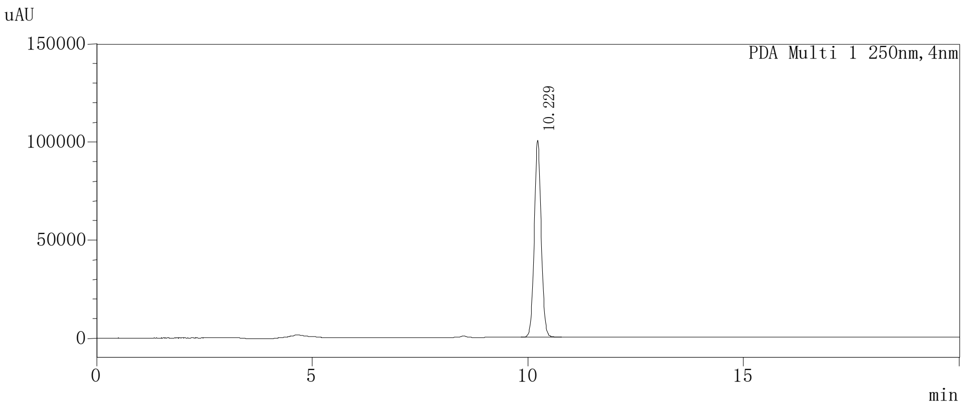

Figure S7. HPLC chromatogram of KG sample (batch no. 8060633)

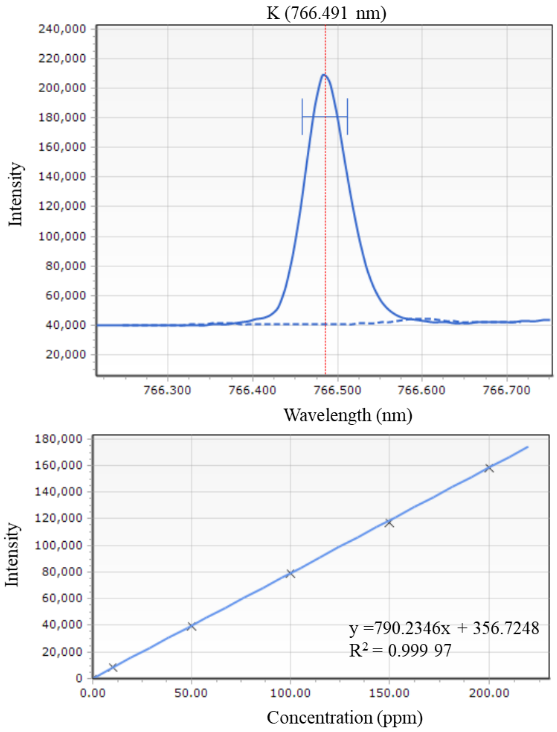

Figure S8. Calibration curve of K by ICP-OES.

Table S1. The integral areas of the internal standards (KHP and FA) and KG at different D1.

D1(s)	A_{KHP}	A_{FA}	A_{KG}
1	2.02	1.69	1.76
2	2.02	1.68	1.69
4	2.00	1.68	1.68
8	2.01	1.75	1.71
16	2.00	1.77	1.71
32	2.00	1.79	1.70
48	2.01	1.79	1.70
64	2.00	1.79	1.70
70	2.01	1.79	1.70

Table S2. Information on ICP-OES operating conditions.

Parameter	Value
Plasma gas flow	12.0 L/min
Auxiliary gas flow	1.0 L/min
Nebulizer gas flow	0.7 L/min
RF power	1200 watts
Plasma view	radial
Read delay	12 sec
Peristaltic pump flow	12 rpm
Spray chamber type	Cyclonic double-pass spray chamber
Nebulizer	Sea spray
Torch	Quartz
Wavelengths(nm)	766.491nm
Number of replicates	3
Observation height	8mm
